# A case of massive hematoma: reflections on hypermobile Ehlers-Danlos syndrome

**DOI:** 10.3389/fmed.2025.1514349

**Published:** 2025-01-28

**Authors:** QingHua Liu, Ganhua Zeng, Yu Xiong, Chenyang Xu

**Affiliations:** People’s Hospital of Ganzhou City, Jiangxi Province, Ganzhou, China

**Keywords:** pulmonary hematoma, case report, hypermobile Ehlers-Danlos syndrome (hEDS), hemoptysis, thoracic surgery

## Abstract

Ehlers-Danlos Syndrome (EDS) refers to a group of connective tissue disorders characterized by significant clinical and genetic variability, affecting multiple systems in the body. Classified as a rare disease, EDS includes 14 subtypes, all marked by joint hypermobility, skin extensibility, and tissue fragility. These subtypes present with a wide range of clinical manifestations and severities, including frequent joint dislocations, scoliosis, arterial dissections, and organ ruptures. Hypermobile EDS (hEDS) is the most common subtype, with newly established clinical diagnostic guidelines. In this case, a patient presented with minor hemoptysis over 8 h, and a chest CT scan revealed a massive hematoma in the left lower lung. Due to the complexity and varied presentations of EDS, misdiagnosis is common. This report shares our experience with diagnosis and treatment in this case, highlighting the importance of increasing awareness for improved survival outcomes.

## 1 Introduction

Hypermobile Ehlers-Danlos Syndrome (hEDS) was classified as Type V in the EDS classification system published by the International EDS Consortium in 2017 (1). The primary diagnostic criterion for hEDS is generalized joint hypermobility, evaluated using the Beighton score (2) (Table 1), with age-adjusted cutoffs. However, a diagnosis of hEDS requires more than just generalized joint hypermobility. At least two additional criteria must be met: systemic connective tissue disease features (such as soft skin, recurrent hernias, spider-like fingers, or mitral valve prolapse), a family history of hEDS, and musculoskeletal manifestations (such as joint instability, frequent dislocations, or chronic pain). Other EDS subtypes and causes of generalized joint hypermobility must also be ruled out (Table 2). The experience in managing skeletal complications in EDS patients is limited, with almost no established guidelines Most of the literature consists of case reports or small retrospective studies (3, 4). In this case, we present a patient with hEDS who developed a hematoma in the left lung. However, the clinical presentation was only mild hemoptysis, which is previously unreported. We will provide insights on the management of this condition.

**Table 1:** Description of subtypes of EDS comparing 2017 nomenclature with the Villefranche and Berlin nosology.

Molecular biology of Ehlers-Danlos syndromes						
Gene	Mutation(s)	Inheritance	Molecular biology	2017 EDS nomenclature	Villefranche nosology	Berlin nosology
ADAMSTS2	Homozygous nonsense mutations [p. (Gln225) and p. (Trp795*)], skipping in frame and out of frame of various exons, homozygous loss of function mutations [c.2927_2928delCT, p. (Pro976Argfs*42); c.669– 670dupG, p. (Pro224Argfs*41); c.2751–2A > T] and compound heterozygous [p. (Met295Thrfs25*)]	AR	Enzymatic component of the ECM	Dermatosparaxis EDS (dEDS)	Dermatosparaxis type	Type VIIC
AEBP1	Compound heterozygous (c,1470delC [p. Asn490_Met495delins(40)] and c,1743C > A [p.Cys581*]), homozygous (c,1320_1326del [p.Arg440Serfs*3]) homozygous splice site variant (c.1630 J)1G > A), homozygous c,1925 T > C p. (Leu642Pro)	AR	Regulates collagen fibrillogenesis	Classic-like EDS (clEDS)	N/A	N/A
B3GALT6	Missense and frameshift mutations, in-frame deletions, start codon mutations, splice site mutations and in-frame duplication. Homozygous and compound heterozygous mutations. The following are the most commonly reported: p. (Pro67Leu), p. (Thr79Ala), p. (Arg232Cys), p. (Asp207His), p. (Phel86Leu), p. (Arg6Trp) p. (Glu265Asp), p. (Ser309Thr), p. (Glul74Alafs*266), p. (Metl?)	AR	Post-translational modification of proteins including proteogylcans	Spondylodysplastic EDS (spEDS)	EDS progeroid type	N/A
B4GALT7	c.808C > T, p. (Arg270Cys); c,122 T > C, p. (Leu41Pro); c.421C > T, p. (Argl41Trp); c.557C > A, p. (Alal86Asp); c.617 T > G, p. (Leu206Pro); c.641G > A, p. (Cys214Tyr); c.277dup, p. (His93Profs*73); c.970 T > A, p. (Cys324Ser)	AR	Post-translational modification of proteins including proteoglycans	Spondylodysplastic EDS (spEDS)	EDS progeroid type	N/A
C1R	Heterozygous missense and in-frame insertion/ deletion variants; p.Asp290Gly, p.Gly297Asp., p.Leu300Pro, p.Arg301Pro, p.Tyr302Cys, p. Ile306_Cys309delinsArgArg p.Cys309Trp, p. Cys309Trp, p.Cys338Arg, p.Cys358Phe, p. Trp364Cys, p.Cys371Trp, p. Arg401_Tyr405delinsHisValIle, p.Trp435Arg	AD	Complement immune system	Periodontal EDS (pEDS)	EDS periodontitis	Type VIII
CIS	Heterozygous missense and in-frame insertion/deletion variants p.Cys294Arg, p.Val316del	AD	Complement immune system	Periodontal EDS (pEDS)	EDS periodontitis	Type VIII
CHST14	Loss of function, missense, frameshift and nonsense mutations p. (Pro281Leu), p. (Val49*), p. (Arg213Pro), p. (Tryr293Cys), p. (Arg29Gfs*113), p. (Lys69*), p. (Glnl 13Argfs*14), p. (Argl35Gly), p. (Leul37Gln), p. (Cysl52Leufs*10), p. (Phe209Ser), p. (Arg218Ser), p. (Gly228Leufs*13, p. Glu262Lys), p. (Arg274Pro), p. (Met280Leu), p. (Cys289Ser), p. (Trp327Cfs*29), p. (Glu334Glyfs*107)	AR	Post-translational modification— conversion of sulfate to dermatan sulfate	Musculocontractural EDS (mcEDS)	N/A	N/A
C0L1A1	c.934C > T, p. (Arg312Cys); c,1720C > T, p. (Arg574Cys); and c.3277C > T, p. (Argl093Cys)	AD	Structural ECM component	cEDS with vascular fragility, vEDS	Classical type, vascular type	Typel/II/IV
C0L1A1	Splice site mutations that lead deletions of exon 6 (intron 5–2A > G/T; intron 5-lG > A/C/ T; exon 6-lG > A/C)	AD	Structural ECM component	Arthrochalasia EDS (aEDS)	Athroscorasia type	Type VIIA/B
C0L1A2	c.213dupC,p. (Arg99*) homozygous, six splice site mutations [two homozygous (c.3105+ 2 T > C and c.3601G > T)] and two compound heterozygous (c.70 + 717A > G; c.1404 + 1G > A and c.540 + 5G > A; c,1404G > C)	AR	Structural ECM component	Cardiac-valvular (cvEDS)	N/A	N/A
	Splice site mutations that lead deletions of exon 6					
C0L1A2	(intron 5–2A > G; intron 5-lG > A/C; exon 6-lG > A; intron 6 + lG > A/T/C; intron 6 + 2 T > C/G)	AD	Structural ECM component	Arthrochalasia EDS (aEDS)	Athroscorasia type	Type VIIA/B
C0L3A1	p. (Gly637Ser)	AD	Structural ECM component	Hypermobile EDS (hEDS)? One Family	Hypermobility type (EDS-HT)	Type III
C0L3A1	Glycine substitutions, splice-site insertions/ deletions, in- frame insertions/deletions, haploinsufficiency, nonglycine missense variants in the triple helix, nonglycine missense variants and in-frame insertions/ deletions, in the N- or C- terminal	AD	Structural ECM component	Vascular EDS (vEDS)	Vascular type	Type IV
C0L5A1	Mutations leading to nonsense mediated mRNA decay, haploinsufficiency and structural mutations	AD	Structural ECM component	Classical EDS (cEDS)	Classical type	Type I/II
COL5A2	Structural mutations and splice site mutations	AD	Structural ECM component	Classical EDS (cEDS)	Classical type	Type I/II
C0L12A1	Heterozygous missense mutations that are autosomal dominant [c.7167 T > C, p. (Ile2334Thr), C.C5893T, p. (Argl965Cys), 8329G > C, p. (Gly2777Arg), C.G8357A, p. (Gly2786Asp)] and a homozygous frameshift mutation that is autosomal recessive (c.8006 + 1 G > A), p. (2567Asp > Phefs*2)	AR or AD	ECM component	Myopathic EDS (mEDS)	N/A	N/A
DSE	Homozygous loss of function, missense mutations [p. (Arg267Gly), p. (Ser268Leu)]	AR	Biosynthesis of dermatan sulfate	Musculocontractural EDS (mcEDS)	N/A	N/A
FKPB14	Duplication [c.362dup, p. (Glul22Argfs*7), homozygous deletion (c,197 + 5_197+ 8delGTAA)], compound heterozygous mutations	AR or compound heterozygosity	ER folding/ transport of collagen	Kyphoscoliotic EDS (kEDS)	Kyphoscoliosis type	Type VI, Type VIA
LZTS1	p. (His211Gln)	AD	Tumor suppressor	Hypermobile EDS (hEDS)? One Family	Hypermobility type (EDS-HT)	Type III
PRDM5	c. 1517_1527dell 1, p. (Val506Glufs*5); c.974delG, p. (Cys325Leufs*2); c.711_714delTGTT, p. (Val238Alafs*35); c,1768C > T, p. (Arg590*); c.320A > G, p. (Tyrl07Cys); c,17 T > G, p. (ValôGly); C.93+ 1G > A	AR	Collagen synthesis	Brittle cornea syndrome (BCS)	N/A	N/A
PL0D1	p.Ile454IlefsX2, p.Ala667Thr, and p.His706Arg, homozygous for exons 10–16 duplication, p. Ile454IlefsX2, homozygous for p.Arg319X	AR	ER folding/ transport of collagen	Kyphoscoliotic EDS (kEDS)	Kyphoscoliosis type	Type VI, Type VIA
SLC39A13	Homozygous 9-bp in-frame deletion in exon 4	AR	Zinc transporter	Spondylodysplastic EDS (spEDS)	EDS progeroid type	N/A
ZNF469	5294delA, 9527delG, Cys 3339Tyr, Glul392Ter	AR	Collagen synthesis	Brittle cornea syndrome (BCS)	N/A	N/A

Gene mutation, inheritance pattern, molecular biology, nomenclature, and references are listed.

**Table 2:** EDS diagnostic checklist listing the three main criterion.

Criterion 1: Generalized joint hypermobility
Beighton score:
≥6 in prepubertal children and adolescents
≥5 in pubertal men and women up to age 50
≥4 men and women over the age of 50
If one point below cut off two or more “yes” answers to the five point questionnaire should be considered.
Criterion 2: Two or more of the following features (A, B, or C) must be present
Feature A (five must be present)
Unusually soft or velvety skin
Mild skin hyperextensibility
Unexplained striae distensae or rubae at the back, groin, thighs, breasts and/or abdomen in adolescents, men or prepubertal women without a history of significant changes in weight
Bilateral piezogenic papules of the heel
Recurrent or multiple abdominal hernias
Atrophic scarring involving at least two sites without the formation of papyraceous and/or hemosideric scars
Pelvic floor, rectal and/or uterine prolapse in children, men or nulliparous women without history of morbid obesity or predisposing medical condition
Dental crowing and high or narrow palate
Arachnodactyly (positive Walker sign or Steinberg sign on both sides)
Arm span to height ratio ≥ 1.05
Mitral valve prolapse
Aortic root dilatation with Z-score > +2
Feature B
Positive family history (one or more first degree relatives meeting current criteria for hEDS)
Feature C (must have at least one)
Musculoskeletal pain in two or more limbs, recurring daily for at least 3 months
Chronic, widespread pain for ≥3 months
Recurrent joint dislocations or joint instability in the absence of trauma
Criterion 3: All of the following must be met
Absence of unusual skin fragility, which should prompt consideration of other types of EDS
Exclusion of other heritable and acquired connective tissue disorders. In patients with an acquired connective tissue disorder, additional diagnosis of hEDS requires meeting both features A and B of criterion 2. Feature of criterion C cannot be counted in this situation

## 2 Case presentation

**Figure 1:**
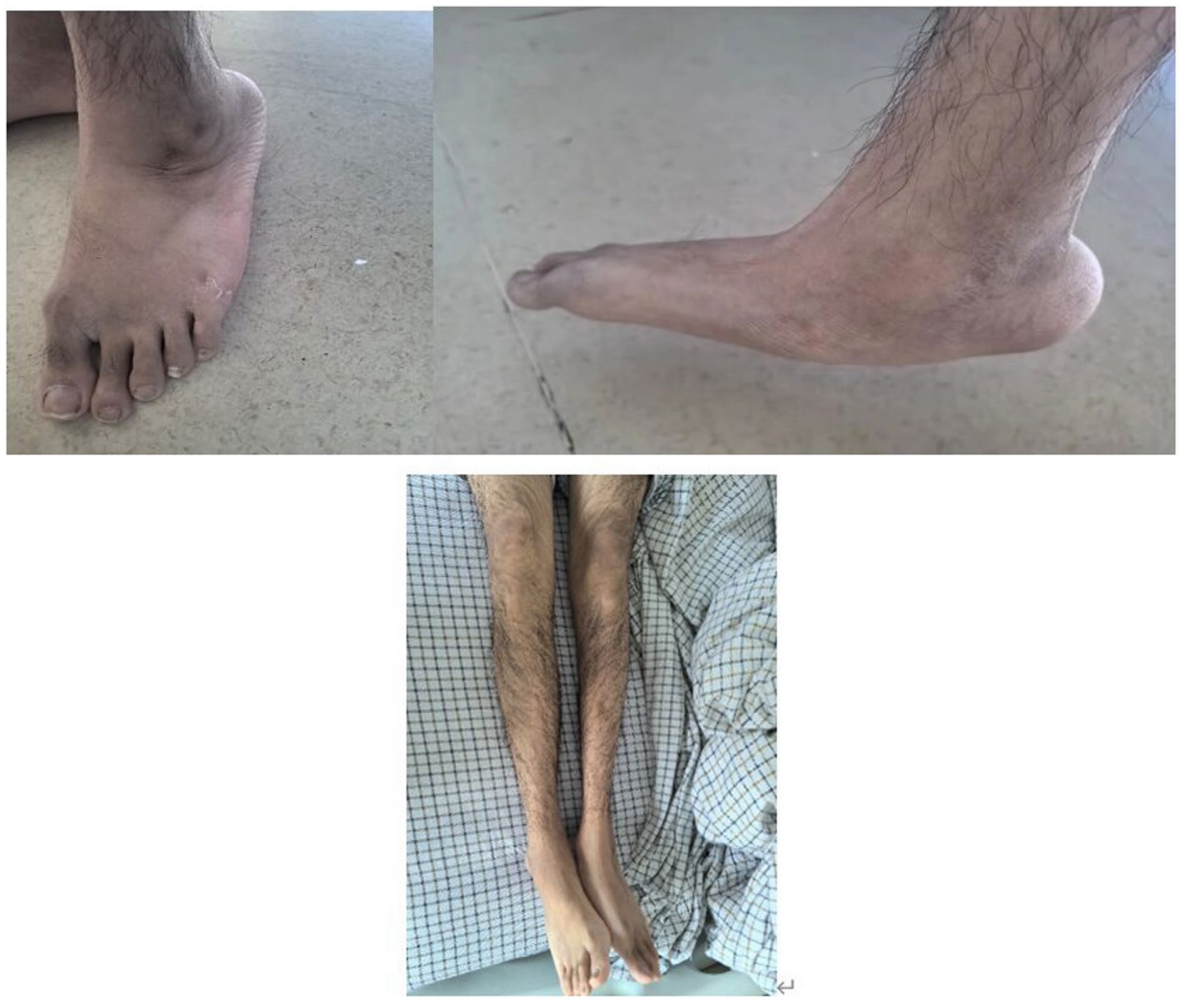
Increased hair on both lower extremities and flatfoot.

**Figure 2:**
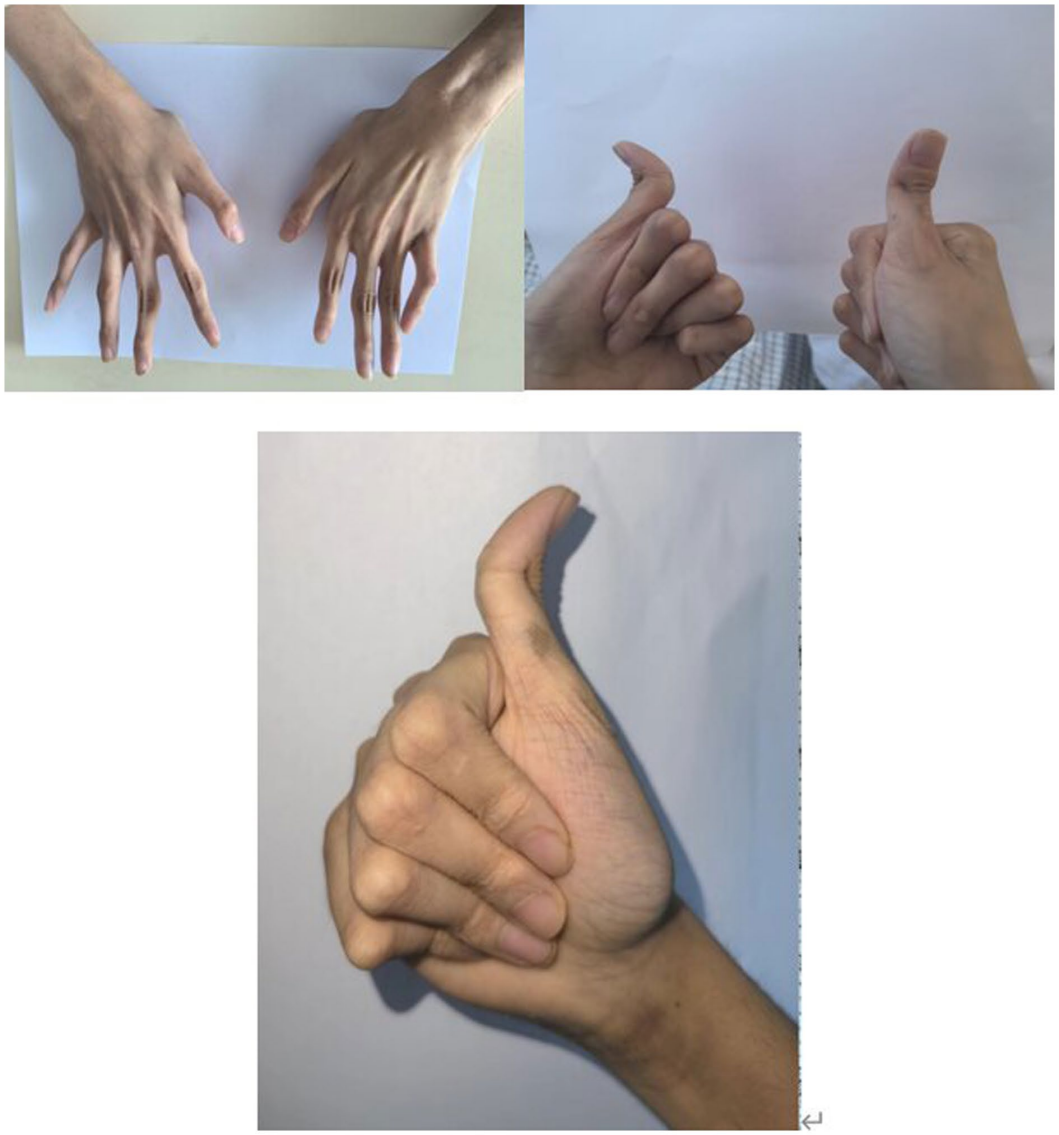
Joint abnormalities and Joint hyperextension.

**Figure 3:**
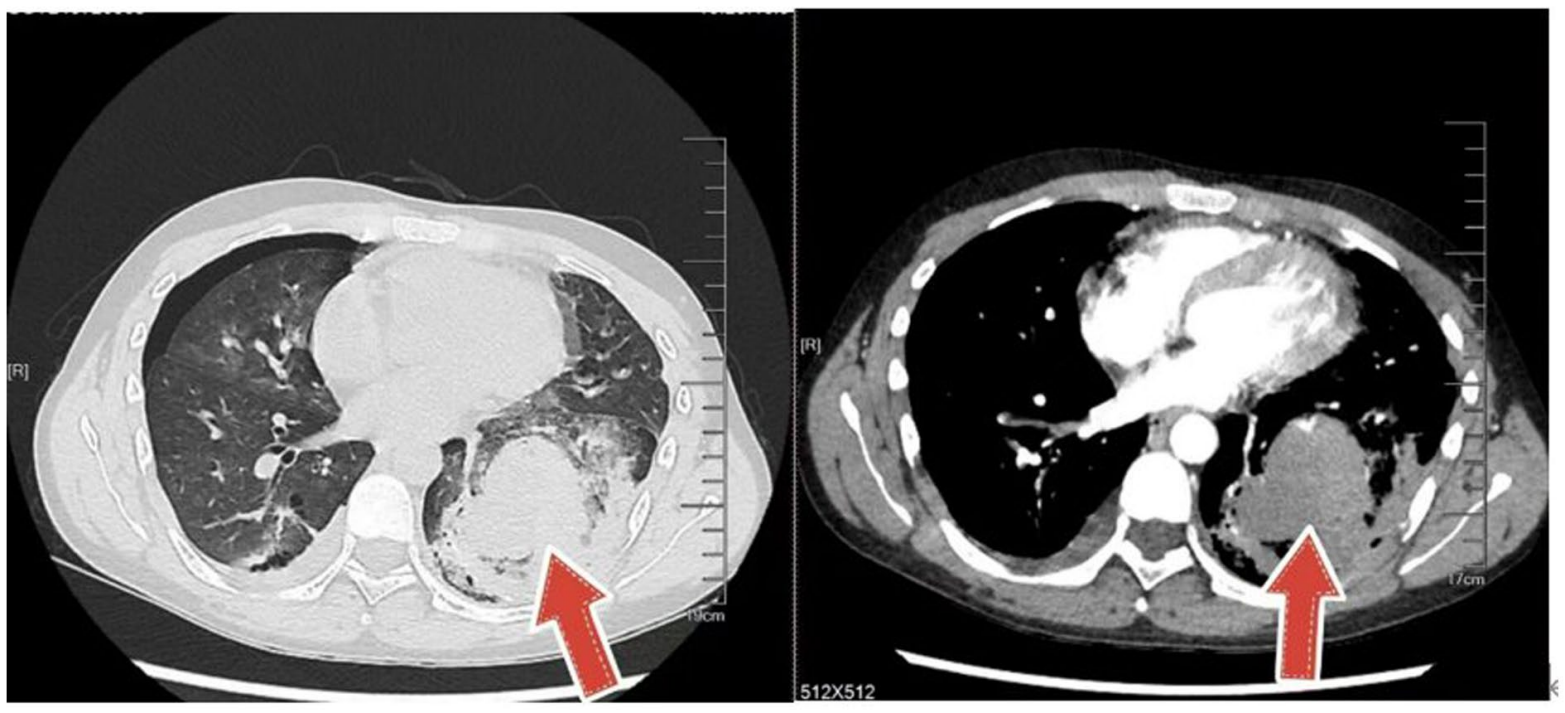
CT shows a massive hematoma in the left lung.

**Figure 4:**
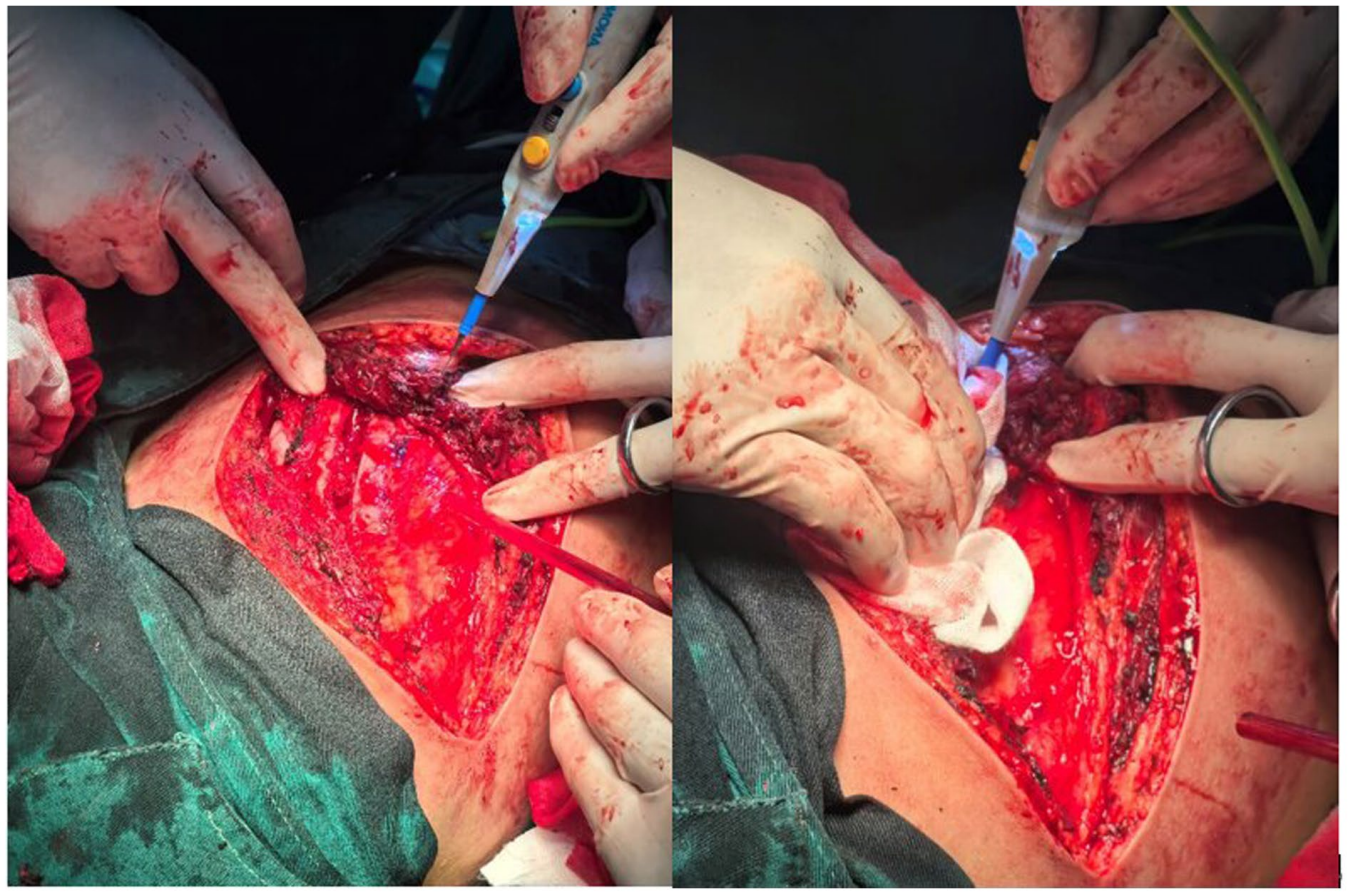
During the surgery, the tissues exhibited abnormal fragility and were prone to bleeding.

An 18-year-old male presented with minor hemoptysis lasting 8 h. Physical examination revealed soft, lax skin, flexible flat feet, hallux valgus, and hypertrichosis (Figures 1, 2). His medical history included spontaneous right pneumothorax, intracranial hemorrhage, spontaneous bleeding in the left calf, and chronic joint pain in the limbs. A chest CT scan revealed a hematoma in the left lower lung (Figure 3). Routine blood tests, biochemistry, and coagulation function were normal (white blood cell count: 10.32 × 10^9, neutrophils: 49.3%, lymphocytes: 29.3%, eosinophils: 8.2%; hemoglobin: 95 g/L, platelet count: 409 × 10^9), and HIV tests were negative. Due to the massive hematoma and persistent hemoptysis, a left lower lobectomy was considered. However, during surgery, the patient’s tissue displayed abnormal fragility, making hemostasis difficult, which prevented a successful lobectomy (Figure 4).

Postoperatively, the unusual connective tissue fragility led to further diagnostic workup, including autoimmune antibody testing, all of which returned negative. The patient’s physical appearance, with visible veins under his translucent skin, combined with a slender build (height: 159 cm, weight: 40 kg, BMI: 15.82), joint hypermobility, and elastic skin, led to a clinical suspicion of EDS. We conducted whole exome sequencing on the patient, but unfortunately, no genetic mutations were identified. However, due to financial constraints, genetic testing was not performed on the patient’s parents. However, we discovered that the patient’s grandfather had a related medical history, and the final diagnosis was hEDS associated with a large pulmonary hematoma. The patient was discharged 2 weeks after surgery. Due to the fragility of the connective tissue, further medical intervention was required. Conservative treatment was implemented, with close monitoring of the patient’s pain management. Physical therapy was provided to help improve joint stability and muscle strength, which is a key aspect of managing hypermobile Ehlers-Danlos syndrome (hEDS). We provided support for unstable joints, including finger splints and ankle stabilizing braces.The patient continued to take nonsteroidal anti-inflammatory drugs (NSAIDs) to manage postoperative chronic pain. Additionally, daily oral vitamin C (100 mg, three times a day) was recommended to enhance vascular elasticity and reduce the risk of potential bleeding.

Upon discharge, the patient was instructed to return for follow-up chest CT scans every 3 months and to avoid prolonged sun exposure. Dietary advice included consuming foods rich in vitamin C. If necessary, the patient was advised to visit the rheumatology department for evaluation of the impact of connective tissue fragility on other organ systems or the orthopedics department to assess the long-term stability and functionality of the joints.

One week after discharge, our department conducted a follow-up with the patient. The patient reported stable condition and significant pain relief compared to earlier. However, no subsequent follow-up visits or evaluations were completed. For a detailed timeline, please refer to Supplementary material.

## 3 Clinical genetics and management discussion

hEDS is a hereditary connective tissue disorder. According to Demmler et al. (5), the prevalence of hEDS and hypermobility spectrum disorders (HSD) in Wales is about 1 in 500, with 70% of diagnosed patients being female. Unlike other EDS subtypes, the genetic cause of hEDS remains elusive, complicating both research and diagnosis. Thirteen of the 14 EDS subtypes have identifiable genetic markers (6), but hEDS remains a diagnosis made based on clinical criteria (Table 3).

In hEDS, connective tissue fragility, particularly in the vascular structures, predisposes individuals to spontaneous bleeding. The underlying pathophysiology involves abnormalities in collagen synthesis, leading to weakened blood vessel walls and an increased tendency for vessels to rupture under minimal stress. This explains the patient’s intracranial hemorrhage and spontaneous bleeding in the left calf.

Pulmonary bleeding, as observed in this case, is less commonly described in hEDS but can also occur due to vascular fragility. The weakened capillaries and small blood vessels in the lungs are susceptible to rupture, especially in the context of trauma or increased intrathoracic pressure. While pulmonary hematoma is a rare complication in hEDS, it should be considered as part of the spectrum of vascular manifestations in this condition.For patients suspected of having EDS, genetic testing is recommended to confirm the diagnosis, though not all patients undergoing clinical diagnosis will have molecular diagnostic results (7). For instance, about 10% of patients clinically diagnosed with classical EDS (cEDS) may not have an identifiable genetic marker (7). Additionally, some patients with mild phenotypes of COL5A1 and COL5A2 mutations may be diagnosed with hEDS rather than cEDS. Some patients exhibit more severe phenotypes or do not completely fit the criteria for cEDS and are clinically diagnosed with vascular EDS (vEDS), dermatospraxis EDS(dEDS), or kyphoscoliotic EDS(kEDS) (8–10). Genetic testing may also reclassify patients diagnosed with EDS into other conditions like Loeys-Dietz syndrome or Cutis laxa or Multiple epiphyseal dysplasia or Hypophosphatasia (7, 11, 12). This is related not only to the strong clinical and genetic heterogeneity of EDS and its intrinsic disease characteristics, but may also be associated with inaccurate phenotype assessment or the absence of complete disease features in younger patients.

The complexity of EDS diagnosis, stemming from clinical and genetic heterogeneity, can lead to uncertainties for clinicians. Negative or ambiguous genetic results do not rule out EDS, which underscores the need for regular re-evaluation of genetic data using advanced methods like RNA sequencing. Studies have shown that regularly re-analyzing genetic data can significantly increase diagnostic accuracy due to evolving knowledge of gene-disease associations and improved phenotypic assessments (13–15).

### 3.1 Management discussion

Pain is the most common symptom in hEDS, and nearly all hEDS patients experience pain, although its severity and frequency can vary widely. Patients with joint hypermobility in EDS may progress from hypermobility to chronic, progressive pain, leading to reduced mobility, decreased muscle mass, proprioceptive dysfunction, and conditions like arthritis. Other common skeletal manifestations include scoliosis (kyphoscoliosis), clubfoot, pectus excavatum or carinatum, osteopenia or osteoporosis, and congenital hip dislocation (16).

One study (17) suggests a multidisciplinary approach for hEDS patients, incorporating medication for pain management, physical therapy, occupational therapy, and psychological support. Medications primarily focus on symptom relief. For example, over-the-counter NSAIDs and analgesics may help alleviate pain and discomfort frequently experienced by EDS patients. However, given that EDS patients often have gastrointestinal issues, medication selection and usage should be guided by a gastroenterologist (18).

Physical therapy serves as a crucial conservative treatment method. It can help enhance joint stability, improve muscle strength, and manage pain.

hEDS is often linked with autonomic dysfunction, and when it impacts the cardiovascular system, it manifests as orthostatic intolerance, including Postural Orthostatic Tachycardia Syndrome (POTS). POTS is defined as an increase in heart rate of at least 30 beats per minute or a heart rate exceeding 120 beats per minute within 10 min of standing, without orthostatic hypotension. However, in this case, POTS symptoms were not observed. Some studies report that the prevalence of hEDS among POTS patients ranges from 15 to 22% (19, 20).

Regarding POTS management (21, 22), early exercise training and increased salt and fluid intake are recommended to expand blood volume. Other non-pharmacological strategies include elevating the head of the bed during sleep, using lower limb compression garments or abdominal binders to reduce venous pooling while standing, and physical maneuvers such as squeezing a rubber ball, crossing legs, muscle pumping, squatting, or negative pressure breathing to prevent orthostatic intolerance and manage acute clinical symptoms in POTS patients. Autonomic dysfunction may also present as gastrointestinal motility disorders, bladder dysfunction, skin discoloration, or abnormal sweating (23).

In terms of the skin, many types of EDS can exhibit skin hyperextensibility, but it is usually less pronounced than in hEDS or cEDS, along with a reduction in dermal thickness and increased skin fragility (24, 25). Patients may experience wound healing defects, potentially leading to atrophic scar formation (25–27). Increased capillary fragility can result in frequent bruising and delayed healing. Oral or intravenous vitamin C can help improve vascular fragility and reduce bruising, but the dose should not exceed 500 mg/day (27, 28). The patient was instructed to take Vitamin C orally, three times a day, 100 mg per dose, upon discharge.

hEDS is frequently associated with symptoms such as fatigue, urticaria, flushing, angioedema, rhinitis, and diarrhea, potentially linked to mast cell activation disorder (MCAD) in hEDS (29). Treatment for MCAD involves avoiding triggers such as certain foods, medications, and temperature changes. Symptomatic treatment includes antihistamines for allergic symptoms, mast cell stabilizers like ketotifen to reduce the release of mediators from mast cells, corticosteroids for acute or severe symptoms to suppress inflammation, leukotriene receptor antagonists like montelukast to alleviate inflammation, NSAIDs for pain and inflammation control, and immunosuppressants in refractory cases (30). Regular monitoring of mast cell mediator levels is also necessary to assess treatment efficacy.

Psychological and mental health management is crucial, not only in hEDS but across all EDS types. Psychological issues such as depression, anxiety, attention deficit hyperactivity disorder (ADHD), and post-traumatic stress disorder (PTSD) are frequently observed in EDS patients (31). Pain is a prominent and pervasive symptom that troubles EDS patients. Research indicates (32, 33) that psychological health is closely associated with pain in EDS, revealing a significant positive correlation between pain intensity and the severity of depressive symptoms. Furthermore, psychological and emotional issues may exacerbate the perception of pain. In this patient, the ongoing involvement of multiple disciplines and frequent hospital visits led to a loss of confidence in the treatment. This required us to provide clear explanations about the condition and offer emotional support.

Physical therapy and medication can improve the patient’s physical condition, while cognitive-behavioral therapy and other psychological interventions can enhance self-efficacy and emotional well-being, helping to reduce fear associated with movement and pain during treatment.

### 3.2 Surgical experience discussion

According to the literature (34), due to the increased vascular and tissue fragility in EDS patients, numerous surgical complications can occur, such as spontaneous expansion of surgical incisions and rupture of deep tissues upon contact with the scalpel. Therefore, extreme caution is required when handling tissues, and the use of skin retractors should be minimized. If the patient has significant vascular fragility, adequate hemostasis through electrocautery should be performed during surgery, and the use of vascular clamps should be avoided. Because the tensile strength of the patient’s blood vessels is poor, arterial repairs can be challenging. Hemostasis can be achieved through gauze compression and intermittent horizontal mattress suturing.When closing the incision, skin closure should be performed in two layers, with minimal tension, sufficient suture material, deep sutures, and three-dimensional support. The sutures should be placed at a proper distance from the incision to avoid cutting through fragile tissues, and skin clamps should not be used. Finally, the duration for retaining the sutures should be twice the usual recommended time to avoid wound dehiscence (35).

## 4 Conclusion

In summary, we have depicted a case of hypermobile Ehlers-Danlos Syndrome (hEDS) presenting as mild hemoptysis, a clinical manifestation not previously documented. This case highlights the importance of considering hEDS in patients with joint deformities and pain. hEDS is a hereditary connective tissue disorder primarily diagnosed through clinical features rather than genetic markers. The patient exhibited tissue fragility during treatment, complicating the surgical process and emphasizing the complexity of the condition. Pain is the most common symptom in hEDS, requiring multidisciplinary management that includes medication, physical therapy, and psychological support. hEDS is also associated with autonomic dysfunction and mental health issues, necessitating psychological interventions to improve quality of life. This case serves as a reminder for clinicians to consider hEDS when encountering joint deformities and unexplained bleeding.

## Data Availability

The original contributions presented in the study are included in the article/supplementary material. Further inquiries can be directed to the corresponding author.
