# Supplementary material for: A case of massive hematoma: reflections on hypermobile Ehlers-Danlos syndrome
Source: Front Med (Lausanne). 2025 Jan 28;12:1514349. doi: 10.3389/fmed.2025.1514349 (PMC11841416; doi:10.3389/fmed.2025.1514349)

GENOWISE
健路医学检验

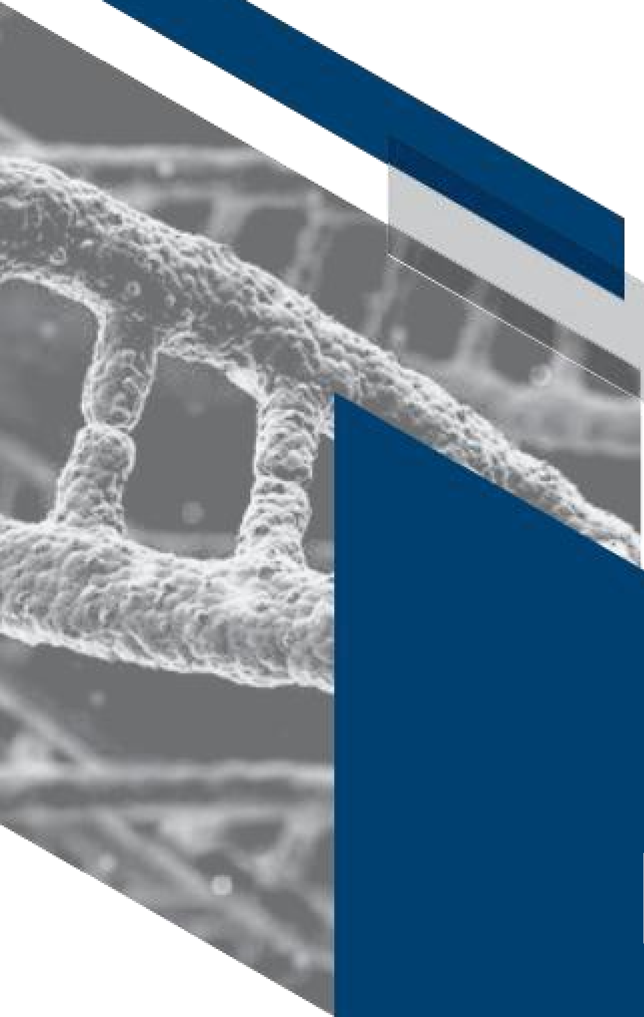

Genetic test

GENETIC TESTING

Contents Content

P 1 essential information

Examinee information

Sample Information

Clinical Information

Testing Program

P 2 Overview of test results

Test conclusion

P 3 ACMG recommends discovery

ACMG Recommended Findings

Introduction to Genes

Disease Introduction

Patient Advice

P 4 appendix

Test Methods and Instructions

Quality Control Information

Related Terms

bibliography

Full exome sequencing test report

essential information

Examinee information

Name： Patient	Gender： Male	Age： 18
Clinical diagnosis： Ehlers-Danlos syndrome （EDS）	Sending unit： -	Sending doctor： -

Sample Information

Barcode number： GW24B229C07	Sample type：Blood	Sampling site:	Pathology number/inpatient number:–
Date of receipt：2024-08-30	Report date： 2024-09-20	Sample status:	Remarks:

Clinical Information

Clinical diagnosis details ： Ehlers-Danlos syndrome （EDS）	
Clinical symptoms/Description	
Family history / past medical history	
Previous surgical or medication history	
Other information<br>**(Gene testing history, etc.)**	

Testing Program

project name	test method	clinical application
Whole exome sequencing	high-throughput sequencing	carrier screening；<br>Genetic disease auxiliary diagnosis；<br>To assist clinicians in understanding the genetic status of patients.

Overview of test results

Testing Conclusion

1. Genotypic related gene variation in the subject

Through high-throughput gene detection technology and bioinformatics analysis, the results showed that:

No pathogenic/potentially pathogenic variation related to the phenotype of the subject was found in the genome of the subject.

2. Other genetic variations associated with hereditary diseases

Through high-throughput gene detection technology and bioinformatics analysis, the results showed that:

No other pathogenic/potentially pathogenic variation was found in the genome of the subjects.

Note:

1. The pathogenicity level of variation is derived from ACMG guidelines and public databases such as Clinvar. With the continuous improvement of relevant guidelines and databases and the update of clinical data,

The classification of variation may change. The clinical significance of genetic variation is divided into five levels according to the relevant ACMG guidelines (VUS sites with unclear clinical significance that do not correspond to clinical phenotype and all possible benign sites Likely Benign and benign sites Benign are not listed in the report):

- Pathogenicity grade Pathogenic;
- Possible pathogenicity grade Likely Pathogenic;
- Clinical significance is not clear grade VUS;
- Possible benign grade Likely Benign;
- Good grades Benign.

2. Special symbols for disease names
   - [ ]: Not a disease, but a variation that causes abnormal laboratory indicators (such as protein deficiency hyperthyroidism);
   - { }: Genetic susceptibility to multiple-factor diseases (such as diabetes, asthma) or susceptibility to infections (such as malaria);
   - ?: The relationship between genes and disease is temporary.

3. Mode of inheritance
   - AD: Autosomal Dominant Inheritance, autosomal dominant inheritance, abbreviated as AD;
   - AR: Autosomal Recessive Inheritance, autosomal recessive inheritance, abbreviated as AR;
   - XLD: X-linked Dominant Inheritance, X-linked dominant inheritance, abbreviated as: XLD;
   - XLR: X-linked Recessive Inheritance, X-linked recessive inheritance, abbreviated as: XLR;
   - YL: Y-linked Inheritance, Y linkage inheritance, abbreviated as: YL;
   Mitochondrial Inheritance, mitochondrial inheritance;
   - DD: Digenic dominant, two genes dominant;
   - DR: Digenic Recessive, two gene recessive;
   - SMu: Somatic mutations, somatic cell mutation.

4. Refer to the version number of the human genome: GRCh 37 (hg19).

ACMG recommends discovery

ACMGrecommenfindings

Through high-throughput gene detection technology and bioinformatics analysis, the results showed that no pathogenic/potentially pathogenic variants of 81 genes recommended by ACMG were found in the genomes of the subjects.

Note:

1 , For general physicians who receive specimens, unexpected findings unrelated to the patients chief complaint generally do not constitute a cause of illness for the patient. Listing this information in the report may unnecessarily worry and anxiety the patient. Therefore, in 2013, the American College of Medical Genetics and Genomics

The ACMG has released a minimum list of genes to be reported as incidental or secondary findings (Secondary Findings, SF). The aim is to identify and manage the risk of selected highly penetrant genetic disorders through established interventions designed to prevent or significantly reduce morbidity and mortality. Subsequently, ACMG established the "Second Survey Results Maintenance Working Group" to develop a procedure for systematically organizing and updating the list. The updated minimum list of secondary findings includes 59 genes recommended for clinical genome sequencing. In May 2021, ACMG noted,

The SF gene list is updated annually, with the version number changed to indicate the updated version and its importance. The latest version is currently named ACMG SF V 3.2. In the latest version update recommendation, the gene list currently includes 81 genes : ACTA 2, ACTC 1, ACVRL 1, etc

APC、APOB 、ATP 7B 、BAG 3 、BMPR 1A 、BRCA 1 、BRCA 2 、BTD、 CACNA 1S、 CALM 1 、 CALM2、 CALM3 、 CASQ 2 、 COL 3A1 、 DES、DSC 2 、DSG 2 、DSP、ENG 、FBN 1 、FLNC、 GAA 、 GLA 、HFE、 HNF 1A 、 KCNH2 、 KCNQ 1 、 LDLR 、 LMNA 、 MAX、 MEN 1 、 MLH 1 、 MSH 2 、 MSH 6 、 MUTYH、 MYBPC 3 、 MYH 11 、 MYH 7 、 MYL 2 、 MYL 3 、 NF 2 、 OTC、 PALB 2 、 PCSK 9 、 PKP 2 、 PMS 2 、 PRKAG 2 、 PTEN 、 RB 1 、 RBM20 、 RET、 RPE 65 、 RYR 1 、 RYR 2 、 SCN5A 、 SDHAF 2 、 SDHB 、 SDHC、 SDHD 、 SMAD 3 、 SMAD 4 、 STK 11 、 TGFBR 1 、 TGFBR 2 、 TMEM 127 、 TMEM43 、 TNNC 1 、 TNNI3 、 TNNT2 、 TP 53 、 TPM 1 、 TRDN、 TSC 1 、 TSC 2 、 TTN、 TTR 、 VHL 、 WT 1。

appendix

Testing methods and Instructions

Whole exome sequencing, which can detect more than 20,000 exonic regions in the human genome and interpret the Human Mendelian Genetics Database (OMIM), Human Genome Mutation Database (HGMD) and NCBI Clinvar define more than 3,000 genes related to pathogenicity and more than 4,000 single-gene genetic diseases, which fully meet the clinical and scientific research needs of carrier screening and accurate diagnosis of genetic diseases.

Gene sequencing and report interpretation have certain limitations. If the gene test does not find suspicious positive sites, it may be related to the following factors:

Objective possibility: The subject may not have a genetic cause of the disease (this does not apply to cases where the subject has a clear family history of the disease, or other evidence has been provided that the patient suffers from a genetic disease).

Current limitations in research: This gene sequencing and report interpretation only targets genes known to be associated with diseases or potentially related to diseases. All data interpretations are based on current understanding of disease and causative genes. Genes not yet studied by this research cannot be interpreted; the report will not include synonymous mutations, non-splicing sites in intron regions, gene regulatory areas, or common benign polymorphic mutations. Pathogenic variations that may exist in these regions cannot be analyzed unless there are existing reports of pathogenicity.

Limitations of the detection method: This sequencing result does not include gene structural variations (such as large deletions, duplications, dynamic mutations, and inversions or rearrangements), large heterozygous insertions, and variations located in gene regulatory regions or deep introns; the DNA used in this method comes from the peripheral blood of the tested individual, not from germ cells, so it cannot rule out interpretation bias caused by chimerism; this detection technique cannot fully cover highly repetitive, highly complex regions, or pseudogenes.

Test results indicate

The results of this report are only responsible for the samples submitted for testing, and all the conclusions are derived from the most advanced scientific research progress in the world.

This test report is only for reference in the clinical diagnosis of the patients related diseases. The clinician should comprehensively consider the applicable information of the test content and combine the actual situation of the patient and other test results (histopathology, imaging) to formulate a comprehensive treatment plan.

Test person: [illegible]　　　　reviewer: [illegible]

健路医学检验实验室
报告专用章

Testing institution: Jianlu Medical Laboratory

Quality control information

Sample primary quality control

Main sample quality control information			
	mass parameter	numeric value	Quality control standards
Sequencing quality assessment	Base quality Q30 percentage	98.17%	≥90%
	Sequence alignment rate	98.95%	≥90%
	capture rate	73.11%	≥30%
	Average sequencing depth	319.54x	≥100
Overall quality assessment	qualified		

paramete

Base quality Q30 percentage: the percentage of base quality above Q30 (i.e., error rate below 1 per thousand) in sequencing data.

Sequence alignment rate: the proportion of sequences successfully aligned to the reference genome.

Capture efficiency: the proportion of sequences (valid sequences) that are matched to target regions out of all sequences that are matched to reference genomes.

Average sequencing depth: the ratio of the number of base pairs in the target region to the size of the target region genome.

Explanations of Relevant Terms

▶ Explanation of the mode of inheritance

In monogenic diseases, according to the different chromosomes on which the gene determining the disease is located, its inheritance mode is mainly divided into the following:

1. Autosomal Dominant Inheritance (autosomal dominant inheritance), also known as AD or ADH. A trait or genetic disease gene is located on an autosomal chromosome, and its nature is dominant. The diseases caused by such genes are called autosomal dominant inheritance disorders. Symptoms can be exhibited in a heterozygous state; if one parent is affected, they will pass the condition to their children, with equal chances of affecting males and females. Depending on the mode of expression, it can be classified into complete dominance, incomplete dominance, codominance, delayed dominance, irregular dominance, incomplete penetrance, sex-linked dominance, and limited dominance.

2. Autosomal Recessive Inheritance (autosomal recessive inheritance), also known as AR. A trait or genetic disease gene is located on an autosome, and its nature is recessive. The disease caused by this gene is called an autosomal recessive inheritance disorder. In a heterozygous state, the corresponding symptoms do not manifest; only in a homozygous state does it cause disease. A heterozygous individual is a carrier, characterized by both parents being normal, but their children exhibit symptoms, indicating that both parents are carriers of the disease-causing gene, with equal incidence in males and females.

3. X-linked dominant inheritance (X-linked dominant inheritance), abbreviated as XD. The disease-causing gene is located on the X chromosome and is dominant, so it occurs when the heterozygote is present. Its characteristic is that the father does not pass it on to his son, but can be passed on to his grandchildren through his daughter, and his grandchildren have a 50% chance of being affected.

4. X-linked recessive inheritance (X-linked recessive inheritance), abbreviated as XR. The disease-causing gene is located on the X chromosome. In females, it is only pathogenic in homozygous state, and in heterozygous state, it is a carrier. In males, the disease-causing mutation is only pathogenic in hemizygous state. The characteristic of male patients is that they will pass this disease to their daughters but not to their sons.

5. Y-linked inheritance (Y-linked inheritance). When the gene controlling a certain trait or disease is located on the Y chromosome and is passed down with it, this is called Y-linked inheritance. Its characteristics include: all individuals with Y-linked inheritance are male, and the paternal parent passes the disease-causing gene only to all sons, while daughters remain unaffected, meaning it is a male-to-male transmission, also known as complete male inheritance. Y-linked genes are relatively few and mostly related to testicular formation and sex differentiation.

Other genetic diseases are mainly divided into the following:

1. Mitochondrial Genetics (mitochondrial Inheritance). Mitochondria contain their own DNA, which differs from the DNA in the cell nucleus in terms of base composition, specifically with different levels of guanine and cytosine base pairs, and they do not bind to histones, appearing as exposed circular DNA. Additionally, mitochondria have ribosomes that can synthesize proteins and have the ability to replicate themselves. It is generally believed that mitochondria possess a certain degree of genetic autonomy.

2. Two gene dominant (digenic dominant), abbreviated as DD. Two gene dominant inheritance is defined as the heterozygous mutation of two genes.

3. Two gene recessive (digenic Recessive), abbreviated as DR. Two gene recessive inheritance indicates a homozygous or compound heterozygous mutation in one gene and a heterozygous mutation in the second gene.

4. Somatic Mutation (somatic mutations), abbreviated as SMu. Somatic mutations are genetic changes that occur in somatic cells and can be passed on to the offspring of the mutated cell during cell division. Somatic mutations differ from germline mutations, which occur in germ cells (i.e., sperm and eggs). Somatic mutations are typically caused by

environmental factors, such as exposure to ultraviolet light or certain chemicals. Somatic mutations are not acquired by germ cells and cannot be inherited by the offspring of the mutated cell.

▶ Explanation of the type of variation

1. Synonymous Mutation (synonymous mutation): A new codon is produced after a single nucleotide change, encoding the same amino acid, resulting in no protein mutation. During DNA replication, when one base in the DNA strand is replaced by another, it does not affect the structure or function of the translated protein.

2. Missense Mutation (missense mutation): This occurs when a codon that encodes one amino acid is replaced by another, changing the type and sequence of amino acids in the polypeptide chain. The result of a missense mutation often leads to the loss of function of the polypeptide chain, and many protein abnormalities are caused by such mutations.

3. Nonsense Mutation (nonsense mutation): This refers to the alteration of a codon that represents an amino acid, causing it to become a stop codon, which prematurely terminates the peptide chain synthesis. Although nonsense mutations do not cause errors in amino acid coding, the presence of a stop codon in the middle of an mRNA

leads to the premature termination of translation, resulting in an incomplete polypeptide chain.

4. Stop codon loss (nonstop mutation): refers to the mutation of the original stop codon, resulting in abnormal extension of the carboxyl end of the protein.

5. Splicing acceptor (splice acceptor variant): A splicing variant that alters the two base regions at the 3 end of an intron. Mutations in these sequences can result in larger segments of intron DNA being retained in mRNA or the entire exon being spliced out of mRNA. These changes can lead to the production of non-functional proteins.

Introns are separated from their exons by splice sites. Receptor and donor sites associated with splice sites signal to the spliceosome for actual cleavage. These donor or recognition sites are crucial in mRNA processing.

6. Donor (splice donor variant): A splicing variant that alters the two base regions at the 5 end of an intron.

Mutations in these sequences can result in larger segments of intron DNA being retained in mRNA or the entire exon being spliced out of mRNA. These changes may lead to the production of non-functional proteins. Introns are separated from their exons by splice sites. Receptor sites and donor sites associated with splice sites signal to the spliceosome for actual cleavage. These donor sites or recognition sites are crucial in mRNA processing.

7. Spliced mutation (splice region variant): A sequence variant that changes within the region of the splice site, i.e., 1-3 bases within an exon or 3-8 bases within an intron.

8. Frameshift Mutation (frameshift): This refers to the alteration of a reading frame in DNA molecules due to the deletion or insertion of a base at a specific site, leading to a series of codon changes downstream and transforming a gene that originally encoded a certain peptide chain into one that encodes an entirely different peptide sequence. Typically, frameshift mutations cause the genes translation to encounter an premature stop codon, resulting in translation termination and the production of truncated proteins. Transcripts with frameshift mutations may also undergo nonsense-mediated mRNA degradation during translation, thus producing no protein product. If translated, these truncated proteins often fail to function properly or not at all, potentially causing

many genetic diseases depending on the inserted gene. Frameshift mutations inevitably alter protein properties, leading to trait variations; in severe cases, they can cause individual death. The DNA damage caused by

frameshift mutations is generally much greater than that caused by point mutations.

9. In-frame insertion (in-frame insertion): When the reading frame is not altered by an insertion, it is called in-frame insertion. The number of inserted nucleotides can be divisible by three. If the inserted nucleotides do not encode a stop codon, the reading frame will remain intact after insertion, allowing translation to proceed smoothly.

However, due to the insertion of nucleotides, the final protein will contain multiple new amino acids that may affect protein function depending on the size of the insertion.

10. In-frame deletion (in-frame deletion): During gene translation, every three nucleotides form a codon. If an entire codon is deleted while the other codons can still translate normally, this mutation is called an in-frame deletion. Compared to out-of-frame deletions (out-frame deletion), the severity of diseases caused by in-frame deletions is generally milder.

11. 5'UTR: A variant of the UTR. This region is crucial for regulating the translation of transcripts through different mechanisms in viruses, prokaryotes, and eukaryotes. Although referred to as non-translational, the 5 UTR or parts of it can sometimes translate into protein products. These products then regulate the translation of the main coding sequence of the mRNA. However, in many organisms, the 5 UTR does not translate at all but instead forms complex secondary structures that regulate translation.

12.3 UTR: A variant of the 3 UTR. The 3 UTR mutation can be crucial because one change may lead to alterations in many gene expressions. Transcriptionally, the mutation might affect only the allele and physically related genes. However, since 3 UTR binding proteins also play a role in mRNA processing and nuclear export, the mutation could impact other unrelated genes.

reference

1. Richards S, Aziz N, Bale S, Bick D, Das S, Gastier- Foster J, et al. Standards and guidelines for the interpretation of sequence variants: a joint conse nsus recommendation of the American College of Medical Genetics and Genomics and the Association for Molecular Pathology. Genetics in medicine: official journal of th e American College of Medical Genetics .2015;17(5):405-24. Epub 2015/03/06. doi: 10. 1038/ gim .2015.30. PubMed PMID: 25741868

2. Discussion on the standardization of clinical genetic testing reports and consensus of genetic testing industry [J]. Chinese Journal of Medical Genetics, 2018,35(1): 1-8.

3. Zhang J, Yao Y, He H, Shen J. Clinical Interpretation of Sequence Variants. Current protocols in human genetics .2020;106(1):e98. Epub 2020/03/17. doi:10. 1002/ cphg .98. PubMed PMID: 32176464.

4. Riggs ER, Andersen EF, Cherry AM, Kantarci S, Kearney H, Patel A, et al. Technical standards for the interpretation and reporting ofconstitutional copy-number variants: a joint consensus recommendation of the American College of Medical Genetics and Genomics (ACMG) and the Clinical Genome Resource (Clin Gen). Genetics in medicine: official journal of the American College of Medical Genetics .2020;22(2):245-5 7. Epub 2019/11/07. doi: 10. 1038/s41436-019-0686-8. PubMed PMID: 31 690835

5. Brnich SE, Abou Tayoun AN, Cou ch FJ, Cutting GR, Greenblatt MS, Heinen CD, et al. Recommendations for application ofthe functional evidence PS3/BS3 criterion using the ACMG/AMP sequence variant interpret ation framework . Genome medicine .2019;12(1):3. Epub 2020/01/02. doi:10. 1186/s13073-019-0690-2. PubMed PMID: 31892348

6. Collins RL, Brand H, Karczewski KJ, Zhao X, Alföldi J, Francioli LC, et al. A structural variation reference for medical and population genetics . Nature .2020;581(7809):444-51 . Epub 2020/05/29. doi: 10. 1038/s41586-020-2287-8. PubMed PMID: 32 461652

7. Tavtigian SV, Greenblatt MS, Harrison SM, Nussbaum RL, Prabhu SA, Boucher KM, et al. Modeling the ACMG/AMP variant classification guidelines as a B ayesian classification framework. Genetics in medicine: official journal of the American College of Medical Genetics .2018;20(9):1054-60. Epub 2018/01/05. doi: 10. 1038/ gim .2017.210. PubMed PMID: 29300386

8. Riggs ER, Church DM, Hanson K, Horner VL, Kaminsky EB, Kuhn RM, et al. Towards anevidence-based process for the clinical interpretation of copy number variation . Clinical genetics .2012;81(5):403-12. Epub 2011/11/22. doi:10. 1111/j. 1399-0004.2011 .01818.x. PubMed PMID: 22097934.

9. Kearney HM, Thorland EC, Brown KK, Quintero-Ri vera F, South ST. American College of Medical Genetics standards and guidelines for interpretation and reporting of postnat al constitutional copy number variants. Genetics in medicine: official journal of the American College of Medical Genetics .2011;13(7):680-5. Epub 2011/06/18. doi: 10. 1097/ GIM .0b013e3182217a3a. PubMed PMID: 21681106

10. Beijing Clinical Laboratory Center, Beijing Medical Association Laboratory Medicine Branch, Capital Medical University Department of Clinical Laboratory Diagnostics, et al. Beijing Expert Consensus on Standardized Application of High-Throughput Sequencing Technology in Clinical Testing [J]. Chinese Medical Journal, 2019,99(43):3393-3397.

11. Amendola LM, Jarvik GP, Leo MC, McLaughlin HM, Akkari Y, Amaral MD, et al. Performance of ACMG- AMP Variant-Interpretation Guidelines among Nine Laborato ries in the Clinical Sequencing Exploratory Research Consortium. American journal of human genetics .2016;98(6):1067-76 . Epub 2016/05/18. doi:10. 1016/j. ajhg .2016.03.024. PubMed PMID: 27181684

12. Expert consensus on the clinical application of whole genome sequencing in genetic disease detection [J]. Chinese Journal of Pediatrics, 2019,057 (006): 419-423.

13. Lek M, Karczew ski KJ, Minikel EV, Samocha KE, Banks E, Fennell T, et al. Analysis of protein - coding genetic variation in 60,706 humans. Nature .2016;536(7616):285-91. Epub 2016/08/19. doi: 10. 1038/ nature 190 57. PubMed PMID: 27535533

14. Biesecker LG, HarrisonSM. The ACMG/AMP reputable source criteria for the interpretation of sequence variants. Genetics in medicine: official journal of the American College of Medical Genetics .2018;20(12):1687-8. Epub 2018/03/16. doi:10. 1038/ gim .2018.42. PubMed PMID: 29543229

15. Ghosh R, Harrison SM, Rehm HL, Plon SE, Biesecker LG. Updated recommendation for the benignstand-alone ACMG/ AMP criterion . Human mutation .2018;39(11):1525-30. Epub 2018/10/13. doi: 10. 1002/ humu .23642. PubMed PMID: 30311383

16. Abou Tayoun AN, Pesaran T, DiStefano MT, Oza A, Rehm HL, Bie secker LG, et al. Recommendations for interpreting the loss of function PVS 1 ACMG/ AMP variant criterion . Human mutation .2018;39(11):1517-24. Epub 2018/09/08. doi:10. 1002/ humu .23626. PubMed PMID: 30192042

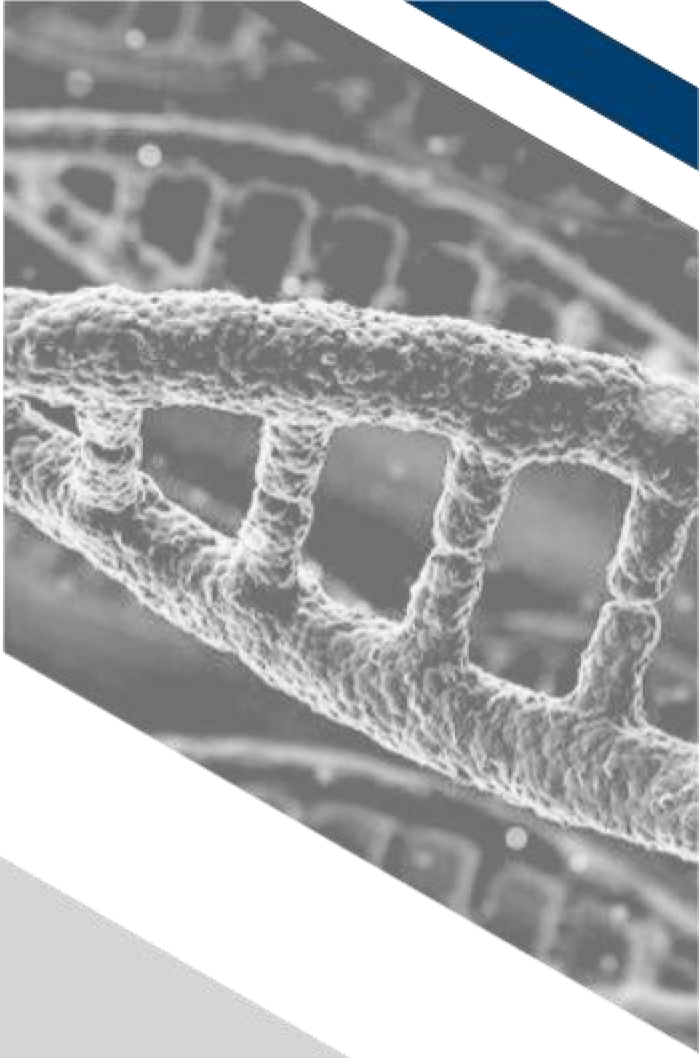

Supplement: Supplementary file 1 [file Supplementary_file_1.pdf]
