# Supplementary material for: A case of massive hematoma: reflections on hypermobile Ehlers-Danlos syndrome
Source: Front Med (Lausanne). 2025 Jan 28;12:1514349. doi: 10.3389/fmed.2025.1514349 (PMC11841416; doi:10.3389/fmed.2025.1514349)
Supplement: Supplementary file 2 [file Supplementary_file_2.docx]

Criterion 1: Generalized joint hypermobility

Beighton score:

> 6 in prepubertal children and adolescents

> 5 in pubertal men and women up to age 50

> 4 men and women over the age of 50

If one point below cut off two or more "yes” answers to the five point questionnaire should be considered (Table 3).

Criterion 2: Two or more of the following features (A, B, or C) must be present

Feature A (live must be piescm):

• Unusually soft or velvety skin

• Mild skin hyperextensibility

• Unexplained striae distensae or rubae at the back, groin, thighs, breasts and/or abdomen in adolescents, men or prepubertal women without a history of significant changes in weight

• Bilateral piezogenic papules of the heel

• Recurrent or multiple abdominal hernias

• Atrophic scarring involving at least two sites without the formation of papyraceous and/or hemosideric scars

• Pelvic floor, rectal and/or uterine prolapse in children, men or nulliparous women without history of morbid obesity or predisposing medical condition

• Dental crowing and high or narrow palate

• Arachnodactyly (positive Walker sign or Steinberg sign on both sides)

• Arm span to height ratio >1.05

• Mitral valve prolapse

• Aortic root dilatation with Z-score >+2

Feature B:

• Positive family history (one or more first degree relatives meeting current criteria for hEDS)

Feature C (must have at least one):

• Musculoskeletal pain in two or more limbs, recurring daily for at least 3 months

• Chronic, widespread pain for >3 months

• Recurrent joint dislocations or joint instability in the absence of trauma

Criterion 3: All of the following must be met

• Absence of unusual skin fragility, which should prompt consideration of other types of EDS

• Exclusion of other heritable and acquired connective tissue disorders. In patients with an acquired connective tissue disorder, additional diagnosis ofhEDS requires meeting both features A and B of

criterion 3. Feature of criterion C cannot be counted in this situation

• Exclusion of alternative diagnoses that may also include joint hypermobility by means of hypotonia and/or connective tissue laxity. Alternative diagnoses may include: neuromuscular disorders (e.g:Bethlem myopathy), other hereditary connective tissue disorders (e.g., Loeys-Dietz syndrome, Marfan syndrome, other types of EDS) and skeletal dysplasias (e.g., osteogenesis imperfecta)
