# Supplementary figures and images for: A case of massive hematoma: reflections on hypermobile Ehlers-Danlos syndrome
Source: Front Med (Lausanne). 2025 Jan 28;12:1514349. doi: 10.3389/fmed.2025.1514349 (PMC11841416; doi:10.3389/fmed.2025.1514349)

# Timeline of the Patient's Care

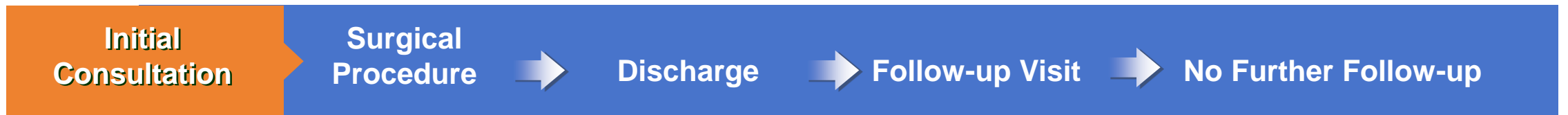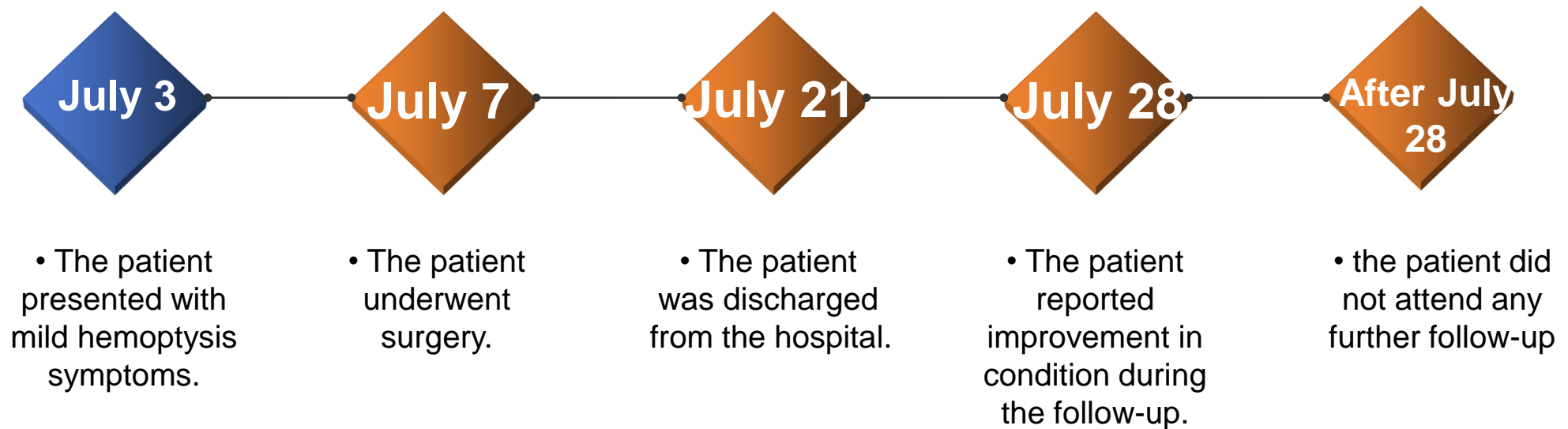

Supplement: Supplementary file 3 [file Data_Sheet_1.pdf]
